# Phase Segmentation in Atom-Probe Tomography Using Deep Learning-Based Edge Detection

**DOI:** 10.1038/s41598-019-56649-8

**Published:** 2019-12-27

**Authors:** Sandeep Madireddy, Ding-Wen Chung, Troy Loeffler, Subramanian K. R. S. Sankaranarayanan, David N. Seidman, Prasanna Balaprakash, Olle Heinonen

**Affiliations:** 1 Mathematics and Computer Science Division, Argonne National Laboratory, Lemont, IL 60439 United States; 2 Department of Materials Science and Engineering, Northwestern University, Evanston, IL 60208 United States; 3 Nanoscience and Technology Division, Argonne National Laboratory, Lemont, IL 60439 United States; 4 Northwestern University Center for Atom-Probe Tomography, Northwestern University, Evanston, IL 60208 United States; 5 Materials Science Division, Argonne National Laboratory, Lemont, IL 60439 United States; 6 Northwestern-Argonne Institute of Science and Engineering, Evanston, IL 60208 United States

**Keywords:** Structural materials, Imaging techniques

## Abstract

Atom-probe tomography (APT) facilitates nano- and atomic-scale characterization and analysis of microstructural features. Specifically, APT is well suited to study the interfacial properties of granular or heterophase systems. Traditionally, the identification of the interface between, for precipitate and matrix phases, in APT data has been obtained either by extracting iso-concentration surfaces based on a user-supplied concentration value or by manually perturbing the concentration value until the iso-concentration surface qualitatively matches the interface. These approaches are subjective, not scalable, and may lead to inconsistencies due to local composition inhomogeneities. We introduce a digital image segmentation approach based on deep neural networks that transfer learned knowledge from natural images to automatically segment the data obtained from APT into different phases. This approach not only provides an efficient way to segment the data and extract interfacial properties but does so without the need for expensive interface labeling for training the segmentation model. We consider here a system with a precipitate phase in a matrix and with three different interface modalities—layered, isolated, and interconnected—that are obtained for different relative geometries of the precipitate phase. We demonstrate the accuracy of our segmentation approach through qualitative visualization of the interfaces, as well as through quantitative comparisons with proximity histograms obtained by using more traditional approaches.

## Introduction

Advances in atom-probe tomography (APT) allow three-dimensional atomic reconstruction of materials with an unparalleled spatial and atomic resolution^1–3^. Applications of APT to a wide variety of materials, for example, inorganic systems such as metals, ceramics, and zeolite-based catalysts^4^, as well as to biominerals and bio-composites composites^5–7^, now provide atomic structure-property relations to facilitate further materials development. APT is particularly useful in studying interfacial properties, for example of precipitates, surfaces and thin films. Once the interface is identified, the elemental distribution along the interface may be closely examined by using various statistical tools, such as proximity histograms^8^ and elemental mapping^9^.

Traditionally, interfaces are identified through iso-concentration surfaces constructed based on the marching cubes algorithm, which extracts an iso-concentration surface from a discrete scalar field with user-supplied concentration values^10^. As the method relies heavily on subjective interface isoconcentration values, it requires an iterative process to determine the precise interface location. In addition, such a labor-intensive manual process prevents analyses of large amounts of APT datasets, limiting the scope of APT studies.

In this paper, we focus on identifying the interface in a precipitate-matrix system by phase segmentation. This approach holds the potential to expedite and reduce inconsistencies in the process of identifying interfaces and study of interfacial properties and furthermore can be scaled up to high-performance computer platforms.

Segmentation is an approach used to partition two- or three- dimensional space into visually distinct and homogeneous regions with respect to certain properties. Segmentation is widely studied in the context of digital images, where the spatial information is represented by means of picture elements (pixels) in two-dimension and volume elements (voxels) in three dimensions.

Classical approaches to segmentation include those based on intensity-thresholding based, edge or boundary-detection based, region/similarity, clustering and graphs approaches (see^11^ for a detailed review). These approaches are primarily unsupervised; the segmentation models are obtained from datasets consisting of image data without any explicitly labeled segments. Several supervised segmentation approaches also exist that use a priori knowledge involving the ground truth of the segments to recognize and label the pixels in a new image according to one of the object classes on which the model is trained. This approach is usually referred to as semantic segmentation^12^ and tends to identify different objects present in an image as well as their location.

The success of deep learning approaches in surpassing human-level accuracy in tasks such as images classification^13^ and language translation^14^ has motivated several recent works in the field of digital image segmentation. This research typically has focused on the semantic segmentation approach, since it has the potential to achieve complete image/scene understanding, which is a crucial aspect of computer vision. There are several applications under the umbrella of computer vision, such as autonomous transport^15^ and human-computer interaction, as well as other applications, such as medical image analysis^16^ and remote sensing^17^, that have adopted semantic segmentation. One shortcoming of this approach is that it is applicable for segmenting only the objects (with a distinct shape) used in training sets.

Alternatively, edge (contour) detection has been significantly improved with deep learning approaches^18^. A supervised learning approach is used for edge detection, wherein each pixel is labeled as either edge or nonedge. This approach is slightly different from semantic segmentation in that there are only two classes (edge and noedge) and less semantic knowledge; hence this approach could be applicable for segmentation of objects with morphologies different from those in the training data. In the case of APT, the precipitate shape can range from a thin slab to a complicated irregular volume; it is therefore a compelling case for the application of edge detection to segment the precipitate from the matrix.

Early deep learning approaches for edge detection used a conventional convolutional neural network (CNN)^19^. Later approaches replaced the CNN with fully convolutional networks (FCNs), which provide an end-to-end framework for pixelwise label prediction^20^. The holistically nested edge detection (HED) approach^21^ was subsequently proposed, which utilizes FCN along with the side outputs (model predictions at the intermediate layers of the network) and deep supervision to significantly improve the edge detection. HED was also demonstrated to achieve human-level accuracy in edge detection. Several enhancements have been proposed for HED^22,23^, but it remains the most widely used approach because of its efficiency and multiscaling scheme to handle resolution and scale problems^18^; hence, we adopt HED for segmentation in this work.

Although deep learning approaches have been shown to be successful in many tasks, one shortcoming is that they rely heavily on being provided with precise and abundant data to train the deep neural networks underlying the approaches. Especially for the supervised learning used for edge detection, large amounts of labeled data are required. Although labeled data are abundantly available for edge detection in natural images, collecting such data is challenging in problems such as interface detection in APT because significant time and effort are required to conduct each experiment^2^ and to manually identify the iso-concentration surfaces as labeled data. We circumvent this challenge of labeled data collection for training the edge detection model by adopting transfer learning, which in general seeks to generalize a model trained on one task to another similar task. More specifically, the transfer learning approach we adopt utilizes the knowledge acquired from learning edge detection features on the source domain (natural images), which has abundant labeled data, for a target domain (APT) edge detection. This transfer learning approach can also be readily generalized to other imaging techniques, such as analysis of x-ray or transmission electron microscopy tomography, as well as to the analysis of synthetic data obtained from, e.g., molecular dynamics simulations, where edge detection of features such as grain boundaries is a central component.

In our work, we present a digital image segmentation based surface extraction as an alternative to manual and, ad hoc construction of iso-concentration surfaces for APT datasets. We show that our approach can transfer learn edge detection features from natural images to segment APT reconstructions accurately and efficiently. We demonstrate the qualitative and quantitative accuracy of our approach using a synthetic dataset constructed with molecular dynamics (MD) simulations, as well as experimental APT datasets of Co and Al superalloys^24,25^. Co and Al superalloys are both precipitation-strengthened alloy systems. Specifically, Co superalloys are strengthened by Co_3_(Al,W) with the L1_2_ crystal structure in a high volume fraction, while Al superalloys are strengthened by Al_3_(Sc,Zr) L1_2_ in high number density.

## Results

### Segmentation

We evaluate the effectiveness and quantitative fidelity of the proposed supervised edge detection and transfer-learning-based digital image segmentation approach using three interface modalities: (1) layered interface, in which the precipitate and matrix are two different layers separated by a thin interface; (2) isolated interface, in which the precipitate is an ellipsoid embedded in the matrix; and (3) interconnected interface, which is a general case where the precipitate phase exhibits an irregular morphology. The layered interface structures examined are synthetic and were generated by using molecular dynamics simulations or using Cameca’s IVAS software (Visualization, I. I. Analysis software for cameca). The isolated and interconnected precipitates APT datasets were obtained experimentally.

#### Layered structures

**Figure 1:**
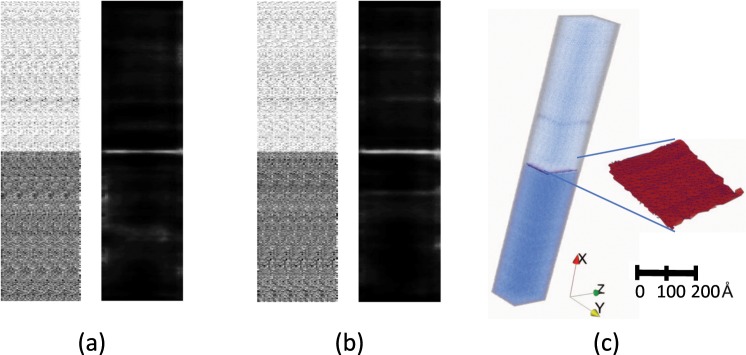
Two- and three-dimensional edge detection map in a synthetic Co-Al alloy generated from MD simulation. (**a**) Concentration space (left) and edge detection map (right) of a slice along the Y-direction, (**b**) Concentration space (left) and edge detection map (right) of a slice along the Z-direction, (**c**) edge surface obtained by merging the edges in the Y- and Z-directions.

A synthetic layer structure was generated by using the MD approach outlined in Sec. 3.4. The Concentration space was obtained for the Co atoms with a voxel size chosen as 2 × 2 × 2 Å^3^. In this particular case, there was no interface component in the X-direction, so only the slices in the Y- and Z- directions were used. A 2D slice in the Y-direction and the corresponding image showing the edge detection map (indicated in white) is shown in Fig. 1(a). The edge detection map correctly identified the interface between the top and bottom regions. A similar exercise on a slice in the Z-direction is shown in Fig. 1(b), where the interface was also captured appropriately by the edge detection map. Merging the 2D edge detection map from slices in the Y- and Z direction, produces the surface separating the top and bottom regions as shown in Fig. 1(c).

**Figure 2:**
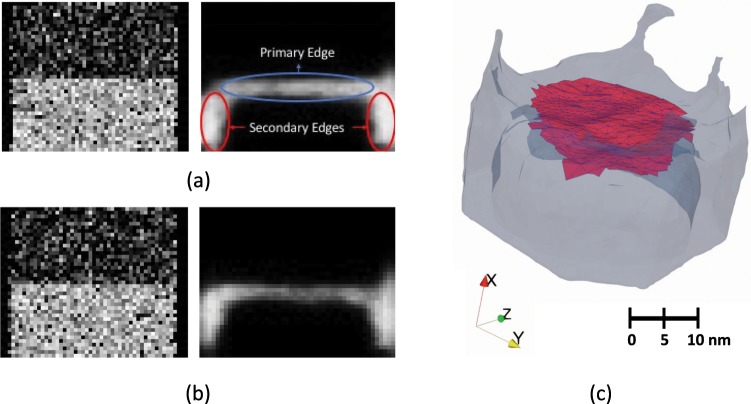
Two- and three-dimensional edge detection map in synthetic Co-Al-W alloy generated by using IVAS. (**a**) Concentration space (left) and edge detection map (right) of a slice along the Y-direction. The desired primary edge separating the two layers as well as the secondary edges is highlighted, (**b**) Concentration space (left) and edge detection map (right) of a slice along the Z-direction, (**c**) edge surfaces extracted after merging the edges in Y- and Z directions.

Similarly, a synthetic structure of a Co-based superalloy generated by using IVAS is shown in Fig. 2. The synthetic structure contains a layer of *γ*′(L1_2_) precipitate and a layer of *γ*(fcc) matrix. This dataset contains additional spatial variations compared with the MD structure. As in the previous case, 2D slices were generated only in the Y- and Z directions to obtain the edge detected surfaces. A slice in the Y-direction and the corresponding edge detection map are shown in Fig. 2(a), and corresponding images for a slice in the Z-direction are displayed in Fig. 2(b). The total number of atoms from all the elements Co, Al and W was 1,983,127, with the dimensions of the enclosing volume being 41 × 51 × 51 nm^3^. The Concentration space was obtained for the Co atoms with a voxel size chosen as 1 × 1 × 1 nm^3^. We note that unlike the previous case, the detected edges are thicker, and we observe secondary edges on the side [Fig. 2(a)]. The thickness of the edges is a consequence of the uncertainty in the boundary between the top and bottom layers as well as the HED edge detection algorithm itself, which tends to produce thicker edges^26^. Other edge detection approaches such as the crisp edge detection (CED)^26^ could be used to alleviate this situation and decrease the thickness of the edges. The consequence of a thicker edge is only that we would obtain two surfaces, one at the top and the other at the bottom as displayed in Fig. 2(c). This does not affect the accuracy of the proximity histogram, or *proxigram* (see Methods section), however, because it shifts the *proxigram* only by a small distance from the interface (see Section 1.2 for a discussion on the *proxigrams* obtained). The secondary edges detected on the side could be removed by preprocessing the 2D slices to remove the empty volume.

#### Isolated phase

To further validate the effectiveness of the implemented segmentation method, we used an experimental APT dataset of a L1_2_ strengthened Al-Er-Sc-Zr-Si superalloy. The interface exhibits a more complex morphology with the precipitate having an ellipsoidal morphology.

**Figure 3:**
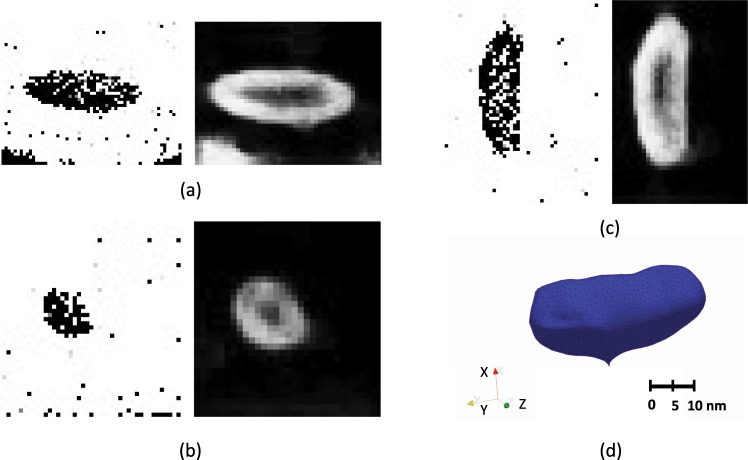
Two- and three-dimensional edge detection map in the experimentally obtained APT data set from an L1_2_-strengthened Al-Si-Sc-Er superalloy. (**a**) Concentration space (left) and edge detection map (right) of a slice along the X-direction, (**b**) Concentration space (left) and edge detection map (right) of a slice along the Y-direction (**c**) Concentration space (left) and edge detection map (right) of a slice along the Z-direction (**d**) the edge surface obtained by merging the edges on slices in the X-, Y-, and Z-direction.

For the APT tomogram with a three-dimensional varying morphology, the 2D slices have to be extracted in each of the three orthogonal directions to reconstruct accurately the precipitate geometry. The 2D slices and the corresponding edge detection map in the X-, Y-, Z-directions are shown in Fig. 3(a–c), respectively. The morphology of the precipitate has been accurately retrieved as seen in the edge detection map obtained on a 2D slice in each of the three orthogonal directions, and the fully reconstructed 3D surface are obtained from fusing all slices in three directions [Fig. 3(d)]. We observe the thick edges in the experimental APT dataset as well. The ability to capture accurately the interface in the Al superalloy, which contains tens of percents of alloying elements (Si, Sc, Zr, and Er), signifies the interface-identifying capability of the HED method.

#### Interconnected phases

**Figure 4:**
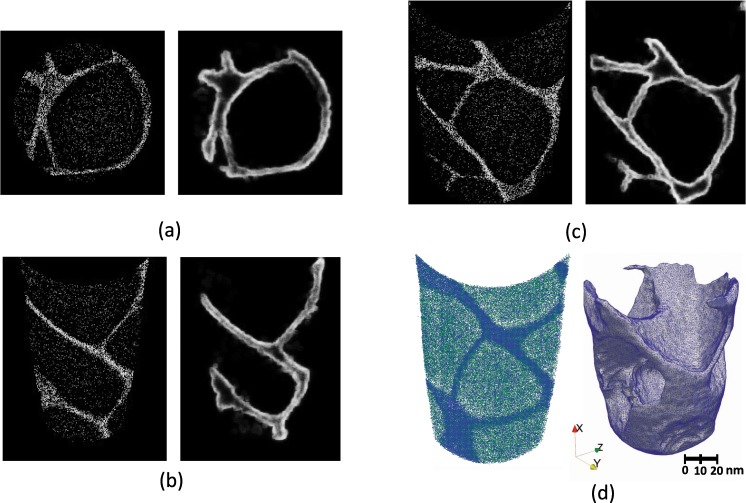
Two- and three-dimensional edge detection map of the experimentally obtained APT data set from a Co-based superalloy. (**a**) Concentration space (left) and edge detection map (right) of a slice along the X-direction, (**b**) Concentration space (left) and edge detection map (right) of a slice along the Y-direction, (**c**) Concentration space (left) and edge detection map (right) of a slice along the Z-direction, (**d**) Three-dimensional point cloud reconstruction of the entire Co superalloy APT specimen (left) and the resulting interface from the edge detection method (right). This method identifies the interconnected *γ* matrix channels on the order of tens of nm.

Figure 4(d) shows the three-dimensional reconstruction of the entire APT nanotip from a Co-based superalloy^24^. The narrow *γ* matrix channel and the interconnectivity of the *γ*′ from precipitate coalescence result in an overall complex interfacial structure.

This is the most general case in which the precipitate is irregular and distributed throughout the examining volume. A 2D slice of the concentration space in the X-direction and the corresponding edges detected are shown in Fig. 4(a), where the edge detection approach accurately identifies and segments the *γ* matrix phase from the *γ*′ precipitate phase. A 2D slice in the Y and Z-directions and their corresponding edge detection map are shown in Fig. 4(b,c), respectively. Figure 4(d) shows the full 3D representation of the surface delineating the *γ* and *γ*′ phases that is obtained by fusing the information from the 2D slices in the three orthogonal directions.

**Figure 5:**
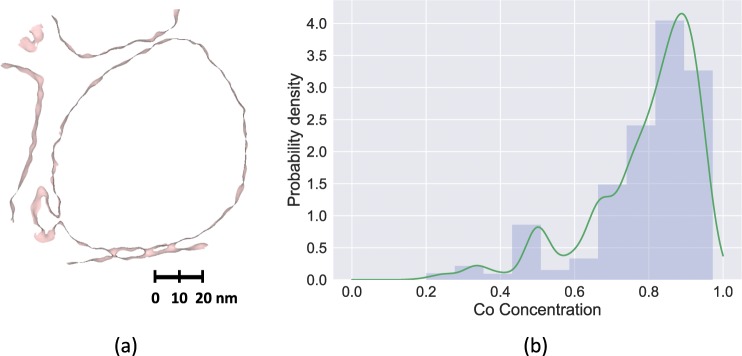
Comparison of iso-concentration interface generated by using IVAS software and the HED-generated interfaces. (**a**) Slice of the iso-concentration surface (Co = 0.855) along the X-axis obtained by using IVAS, (**b**) histogram of the concentrations at the pixels corresponding to the edge detected map shown in Fig. 4(a) (peak at Co = 0.87).

A qualitative comparison of the concentration space and the edge detection maps on each slice shows that the location of the matrix/precipitate interface is fairly well captured by the edges. To make a quantitative comparison, we obtained the iso-concentration surface using IVAS, by carefully tuning the Co concentration until the precipitate and matrix interface is captured. A voxel size of 0.5 nm × 0.5 nm × 0.5 nm was used with 3 nm × 3 nm × 1.5 nm of delocalization via a Gaussian distribution. The obtained iso-concentration surface corresponds to a Co concentration of 0.855, and the detected surface corresponding to this concentration is shown in Fig. 5(a). With the HED approach, for the identical 2D slice, a histogram of the Co concentration at the HED detected edge is shown in Fig. 5(b) and has a peak at Co = 0.87, which agrees well with the iso-concentration surface obtained via IVAS, thus validating the interface detected by using the HED method against the ubiquitous iso-concentration surface approach.

### Proxigram estimation

Interfacial properties of a material are frequently extracted by fitting various models to the *proxigram* composition profile. For example, sigmoid functions are well suited to model a symmetric interface^27,28^ while manual thresholding more accurately extracts the properties of an asymmetric interface^29^. The accuracy of the interfacial properties impacts derivative material properties, such as interfacial free energy. Specifically, the interfacial free energy depends on the mean precipitate radius and the supersaturation of each element in the system, properties that APT datasets aptly capture. However, any interface identification or analysis requires determining the interface location through an iso-concentration analysis. Using the HED approach described here, we performed interface detection through compositional contrast rather than an arbitrarily defined concentration value. Therefore, the HED is agnostic with respect to models of an interface, to both detection and other properties, such as symmetry or asymmetry.

**Figure 6:**
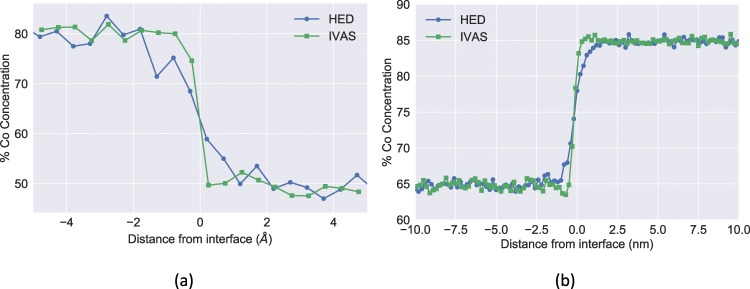
*Proxigram* comparisons on the MD and IVAS generated synthetic datasets. The *Proxigram* concentration profile derived from IVAS display a narrower interfacial compared with the HED method. (**a**) *Proxigram* concentration profile from the synthetic MD layered structure using HED and IVAS, (**b**) *Proxigram* concentration profile obtained from the synthetic IVAS dataset using HED and IVAS.

The resulting *proxigram* from the layered structure generated by using MD is shown in Fig. 6(a), and those of the synthetic IVAS-generated data are shown in Fig. 6(b). For the MD synthetic structure, the HED interface profile is obtained from the time-averaged histograms directly from the MD simulation, whereas the IVAS profile is obtained by importing the MD synthetic structure into IVAS. Because the Co concentration does not monotonically vary across the interface, adopting a spline fit and Co concentration thresholding will more accurately capture the interface properties as commonly practiced in the phase-field modeling community. With the concentration thresholding method, the interface thickness obtained by using the HED method is 2.3 Å, while that obtained from IVAS is approximately 1.3 Å. We note that the interfacial width obtained for the same dataset using the isoconcentration surface obtained from IVAS [Fig. 6(a)] yields an interface thickness that is smaller than that obtained by using the HED approach. The far-field concentrations of Co on either side of the interface (80.2% and 50.7%) are both accurately recovered by the *proxigram* method, which provides a quantitative validation of the interface obtained with our segmentation approach. Far-field concentrations are calculated by averaging the composition after the interfacial contribution has diminished.

Properties of a symmetric interface may be modeled by using the following sigmoid function,

1 $$f(x)=\frac{1}{2}({\rho }_{1}+{\rho }_{2})-\frac{1}{2}({\rho }_{1}+{\rho }_{2})\,\ast \,\tanh \,(\frac{x-a}{b}),$$

where ${\rho }_{1}$, ${\rho }_{2}$, *a*, and *b* are fitting parameters that correspond to the atomic densities of the two regions of the box, the interface position, and the interfacial thickness, respectively. Direct measurement of the interfacial thickness of the MD dataset using Eq. 1 on time-averaged histograms of the Co positions along the interfacial direction results in an interfacial thickness of 3.8 Å, whereas fitting Eq. 1 to the HED *proxigram* results in an interfacial thickness of 4.0 Å. This comparison suggests that the HED interface can closely capture the interface thickness, a capability that is important for calculating material properties that are particularly sensitive to measurement error, such as the coarsening rate constant. We also compare the *proxigram* concentration profile obtained from the synthetic layered data generated by using IVAS [Fig. 6(b)]. In this case, we use the interfacial surface generated by using HED approach as well as that obtained by using IVAS. The *proxigram* concentration profiles generated from IVAS use a bin size of 0.5 nm to average local composition fluctuations. As in the earlier case, we find that the concentrations on either side of the interface are accurately obtained using both approaches. However, even though the interfacial thickness is very close for both approaches, the interfacial thickness obtaind by using the proposed HED approach is slightly larger than than that obtained by using IVAS, consistent with our observations for the synthetic MD-generated interface [Fig. 6(a)].

## Discussion

The ability to both rapidly and accurately identify interfaces, as well as to analyze them qualitatively and quantitatively, is paramount in the analyses of APT data, and also in general to tomographic investigations of multi-grain solid structures^30–33^. Current approaches utilizing iso-concentration surfaces could potentially be scaled on modern architectures but requires subjective input to be accurate.

We have proposed and demonstrated a digital image segmentation approach to segment the precipitate and the matrix phases in superalloys, and we used that approach to obtain the interfaces. The segmentation is accomplished by using supervised edge detection to accommodate the irregularity in the morphologies of the two phases. Specifically, we utilize the hierarchically nested edge detection approach that consists of fully convolutional networks along with deep supervision. Furthermore, because the process of collecting and manually labeling the interface in the APT data for training the edge detection model can be cumbersome, labor-intensive, expensive, and time-consuming, we propose a transfer learning approach to ameliorate this approach. Our approach utilizes the edge detection features learned on the natural images, which have abundant label data, and transfers that knowledge to segmentation in the atom-probe tomography data.

We demonstrated that our approach is qualitatively and quantitatively accurate, by comparing the results of our approach with that of proprietary IVAS software from Cameca Instrument Inc. for synthetic and experimental APT data of two-phase systems. Our approach successfully segmented the two phases and identified the interfaces with different geometrical features. The identified interfaces correspond well with the qualitative visualization using 2D slices. In cases where the images are noisy and the interface is not clear, we obtain *thick* edges, which could be alleviated by using approaches such as crisp edge Detection^26^.

The approach proposed here demonstrates the power of machine learning techniques in the analyses of APT data. It should also be readily applicable to analysis of other tomographic data of multi-phase or multi-grain systems, such as X-ray tomography of multi-phase systems or transmission electron microscopy tomography. By using transfer learning, the fully convolutional network can be trained in advance of experiments and be applied in real time. This may be especially valuable in situations where rapid data analysis during the experiment may provide valuable real-time feedback to the experiment.

## Methods

### Holistically-nested edge detection

We adopt HED, an end-to-end edge detection approach that performs image-to-image prediction (i.e., takes an image as input, and outputs the prediction at each pixel) by means of a deep learning model that leverages FCN and deeply supervised nets. FCN is similar to the regular CNN model used for classification, but the last fully connected layer is replaced by another convolution layer with a large filter size, which allows pixelwise label prediction. Deep supervision is achieved by using the local output from each of the hidden layers (analogous to the final output obtained from a network truncated at the current hidden layer) and back-propagating the error not only from the final layer but simultaneously from all the local outputs in the learning stage. The side outputs and deep supervision contribute to a significant performance gain over the patch-based CNN and simple FCN for edge detection.

The training phase of this approach aims to learn a functional mapping between the two-dimensional input image described by *N* pixels *X*_*k*_, $k=1,\ldots ,N$, where edge detection is desired, and the corresponding ground truth binary edge map (*Y*_*k*_) on all the pixels of image *X*_*k*_. This map is obtained by training a neural network, which is composed of a VGGNet (a neural network consisting of 16 convolutional layers, five pooling layers, and three fully-connected layers, which was proposed by the Visual Geometry Group (VGG)^34^), where the fifth pooling layer and the fully connected layers are trimmed, resulting in five stages with a total of 16 convolutional layers. The side output layer is connected to the last convolutional layer in each stage for deep supervision.

The process of collecting APT data is expensive and time-consuming, and manually identifying the interfaces is cumbersome, labor-intensive and subjective. Hence, we adopt the transfer learning approach in which (1) the parameters for the trimmed VGGNet part of the network are initialized to the weights from VGGNet pretrained on the Imagenet dataset^35^ and (2) the entire network is then trained by using the Berkeley Segmentation Dataset and Benchmark (BSDS 500)^36^ dataset (composed of 200 training, 100 validation, and 200 testing images) containing a wide variety of natural scenes with at least one discernable object (e.g. birds, animals). Each image in the dataset has been manually annotated to obtain the ground truth contours. This training approach is similar to the procedure for edge prediction in natural images outlined by Xie and Tu^21^.

### Orthogonal volumetric segmentation

The neural network trained by using the procedure mentioned in the preceding section is then used to perform segmentation on a three-dimensional APT dataset.

Before segmentation, the data have to be prepared and processed into a suitable format. The datasets obtained from APT consist of the spatial coordinates of each atom and a label for their chemical identity. These data are transformed into a regular 3D cubic grid of atomic concentrations, where the grid is obtained by partitioning the 3D space into a series of cubic voxels (whose dimensions are chosen empirically as is done for traditional interface detection approaches), and concentration is calculated for a given chemical species on each voxel based on its relative atomic fraction with respect to the others. We also note that an additional smoothing function is not used on the obtained concentrations. This grid along with the concentrations is hereafter referred to as the concentration space. The concentration values range between $[0,1]$; hence they can be converted into grayscale images with a single intensity channel, and subsequently replicate these intensity values into three channels corresponding to RGB of each voxel. The segmentation approach can then be applied to the concentration space and its associated RGB voxels.

We propose to segment the concentration space by extracting 2D slices in each of the three orthogonal directions and detecting the edges using the HED model trained on natural images. Once the edges are obtained on each of the image slices in the three orthogonal directions, a 3D edge detection map for each slice direction is obtained by stacking all the 2D edges in that direction. All the 3D edge detection maps obtained from each of the slice directions are then fused into a single 3D edge detection map by combining all the voxels detected as edges in each of the slice direction. In other words, each voxel labeled as the edge in the fused map corresponds to an edge detected in at least one of the slice direction. Since the APT datasets studied here have only two phases, the obtained edge detection map in 3D serves as the interfacial surface between the two phases. We note, however that the thickness of this surface depends on the thickness of the edges delineating the two surfaces. If the edges are thicker and extend to more than one voxel, there could be two surfaces, one on either side of the edge. This configuration is still acceptable, however since it shifts the proximity histograms (described in the Methods section) by only a small distance from the interface and hence does not have significant effects on the interface properties calculations.

### Proximity histogram calculation

The 3D point cloud data from the APT results consist of the atomic positions and elemental identities; when these data are combined with the edge-detected surface, a one-dimensional plot is obtained that represents the change in the relative concentration of the chemical species as a function of the distance from the surface. This plot is referred to as the proximity histogram, or *proxigram*, and is acquired following the procedure outlined in Hellman *et al*.^8^ except that the iso-concentration surface is herein replaced by the interfacial surface obtained by edge detection. Algorithm 1 outlines the approach used to obtain the proxigrams.

### Data acquisition and preparation

#### Synthetic dataset for validation and verification

To create a verifiable test case, we created a synthetic sample with a known concentration using an (MD) approach—an atomistic simulation method, where the motion of atoms is modeled using Newton’s laws of motion. In these simulations, the forces between atoms are modeled using empirical forcefield equations, and motion is created by using various time integration schemes. The synthetic sample was first created by inserting two different amorphous mixtures of Al and Co into a box at opposite ends. The two phases were bridged with an amorphous interface whose mixture was initially a linear gradient of 5 Å. Once the initial structure was generated, it was heated via molecular dynamics using the LAMMPS software package^37^. This heating was done at a constant temperature of 2000 K for 100 picoseconds of simulation time in order to smooth out the interface. The Embedded Atom potential of Pun *et al*. was used for the inter-atomic forces^38^. The MD synthetic structure contains one layer with an 80/20 Co to Al mixture and a second layer with a 50/50 mixture. The total number of atoms from all the elements, Co and Al, was 16,000, and the dimensions of the volume enclosing them were 1000 × 100 × 100 Å^3^.

Similarly, a layered synthetic structure with the composition of a Co-based superalloy was generated by using Cameca’s IVAS software suite. The FCC *γ* layer is generated with a composition of 85% Co, 7.5% Al and 7.5% W, and the L1_2_ ordered intermetallic layer is generated with a composition of 65% Co, 17.5% Al and 17.5% W (in at.%), each with a background of 20 ppm/ns. The detection efficiency is assumed to be 50% as the experimental Co superalloy dataset are obtained from LEAP 4000Si. In order to more closely emulate the structure obtained from APT, where atomic positions deviate from the perfect crystalline lattice due to uncertainties from the field-induced evaporation process^39^, the IVAS synthetic dataset was artificially introduced with a random spatial deviation from the theoretical atomic sites by adding a smear width of 0.15 nm.

#### APT datasets

Cobalt and aluminum superalloys were used as model materials for the experimental APT datasets. The Co-based superalloy used is a ternary alloy with 8.8 at.% Al and 7.3 at.% of W. Coherent precipitates of the *γ*′-phase (L1_2_) are formed in the *γ*-phase (fcc) matrix with concentration differences in the two phases following the bulk thermodynamic potentials. These concentration differences lead to concentration changes across interfaces that can be used to identify them. The total number of atoms from all the elements (Co, Al, and W) is 19,104,918, with the dimensions of the enclosing volume being 112 × 90.5 × 90.5 nm^3^. The concentration space is obtained for the Co atoms with a voxel size chosen as 0.5 × 0.5 × 0.5 nm^3^.

The Al superalloy studied herein is an Al-Er-Sc-Zr-Si alloy that is strengthened by ordered (L1_2_) coherent Al_3_(Er,Sc,Zr) precipitates and has a concentration of 0.005 at% Er, 0.02 at% Sc, 0.07 at% Zr, and 0.06 at% Si. The total number of atoms from all the elements (Al, Er, Sc, Zr and Si) is 668,388 with the dimensions of the enclosing volume being 29.5 × 23.5 × 22.5 nm^3^. The concentration space is obtained for the Al atoms with a voxel size chosen as 0.5 × 0.5 × 0.5 nm^3^.

Atom-probe tomograms of the alloys were obtained by preparing nanotips and were analyzed by using a Cameca’s local-electrode atom-probe (LEAP) 4000X-Si equipped with picosecond ultraviloet laser. During the pulsed laser illumination, surface atoms are evaporated toward a two-dimensional position sensitive detector, thus constructing three-dimensional atomic tomograms of the specimens. Along with the time-of-flight measurements, the mass-to-charge ratio of the atoms can be determined, providing chemical information for each individual atoms. The experimental procedures and tomogram reconstruction conditions are detailed in refs. ^25,40^. The APT raw datasets were processed by using IVAS for mass spectra analyses and spatial reconstructions. After raw data processing, a position file that contains the reconstructed species’ spatial positions and mass-to-charge state (m/n) ratios was obtained. Additionally, a range file that matches an m/n ratio to a particular element enable identification of each atomic species^39^.

**Algorithm 1:**
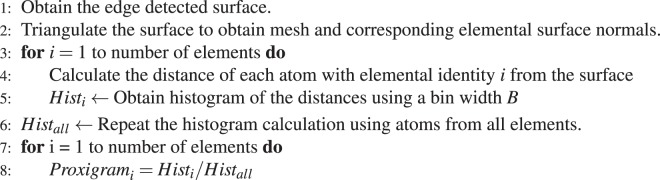
*Proxigram* calculation.

## Data Availability

The datasets generated during and/or analyzed during the current study are available from the corresponding author on reasonable request.
